# Traumatic Brain Injury in the Elderly: Is it as Bad as we Think?

**DOI:** 10.1007/s13670-012-0017-2

**Published:** 2012-07-06

**Authors:** Calvin H. K. Mak, Stephen K. H. Wong, George K. Wong, Stephanie Ng, Kevin K. W. Wang, Ping Kuen Lam, Wai Sang Poon

**Affiliations:** 1 Division of Neurosurgery, Department of Surgery, The Chinese University of Hong Kong, Prince of Wales Hospital, Shatin, Hong Kong; 2 Division of Neurosurgery, Department of Surgery, The Chinese University of Hong Kong, Prince of Wales Hospital, Shatin, Hong Kong; 3 Center for Neuroproteomics and Biomarkers Research, The Department of Psychiatry and Neuroscience, McKnight Brain Institute, University of Florida, Gainesville, FL 32611 USA; 4 Chow Tai Fook-Cheung Yu Tung Surgical Stem Cell Research Center, Department of Surgery, The Chinese University of Hong Kong, Prince of Wales Hospital, Shatin, Hong Kong

**Keywords:** Traumatic brain injury, Elderly, Geriatric, Aging, Review, Animal studies, Stem cell, Drugs, Anticoagulation, Antiplatelet, Warfarin, Aspirin, Clopidogrel, Epidemiology, Outcome, Cognitive, Craniotomy, Decompressive craniectomy

## Abstract

Traumatic brain injury in elderly patients is a neglected global disease burden. The main cause is fall, followed by motor vehicle accidents. This review article summarizes different aspects of geriatric traumatic brain injury, including epidemiology, pathology, and effects of comorbidities and pre-injury medications such as antiplatelets and anticoagulants. Functional outcome with or without surgical intervention, cognitive outcome, and psychiatric complications are discussed. Animal models are also reviewed in attempt to explain the relationship of aging and outcome, together with advances in stem cell research. Though elderly people in general did fare worse after traumatic brain injury, certain “younger elderly” people, aged 65–75 years, could have a comparable outcome to younger adults after minor to moderate head injury.

## Introduction

According to the World Health Organization, traumatic brain injury (TBI) will lead as the major cause of death and disability by the year 2020. It is estimated that 10 million people are affected annually by TBI [1]. Often neglected, elderly TBI patients are going to be an increasing burden to the society with the worldwide aging population. Peschman et al. [2] showed that age alone is associated with increased odds of being admitted to the hospital after head injury.

In the United States, there are 1.4 million cases of TBI per year, causing over 90,000 cases of permanent disability and 50,000 deaths. Specifically, among the elderly, there are 155,000 cases of TBI annually in the United States leading to 12,000 deaths [3]. There is also an increasing incidence of patients with TBI being discharged to the elderly home over the recent years [4].

Elderly patients suffering from head injury were traditionally thought to have an inferior outcome. They were more likely to go to inpatient rehabilitation or long-term care facilities, or died compared to young and middle-aged patients [5]. Elderly TBI patients also bear higher cost during hospital stay in cost-analysis models [6].

Thus, elderly TBI patients tend to be treated less aggressively. In a retrospective multicenter study in Scotland, older patients with acute intracranial hematomas were less likely to be transferred for specialist neurosurgical care than younger patients with similar severities of injuries, extracranial injuries, and physiological status at presentation, irrespective of associated medical morbidities [7].

These concepts are recently being challenged as clinicians observe that with adequate resources, timely and appropriate surgical intervention, neurointensive care, and aggressive neurorehabilitation, both functional and cognitive outcome of elderly TBI patients may be as good as the younger counterparts. We thus reviewed the current evidence in the literature regarding factors affecting outcome after geriatric TBI and aimed to define the role of aggressive neurosurgical management in geriatric TBI.

## Methods

Studies included in the present article were identified by searching through PubMed and MEDLINE using keywords: [traumatic brain injury OR head injury] AND [elderly OR geriatric] AND [epidemiology OR outcome]. Non-English articles were excluded.

## Epidemiology

Trauma literature usually defines “elderly” as more than 65 years of age, which is used in this review unless otherwise specified [6, 7, 8••, 9, 10, 11••, 12–14, 15••, 16–24].

Incidence of elderly TBI has doubled in the past 18 years that the increase in elderly TBI is greatest for individuals aged 83–90 years [15••]. Fall at level ground is the leading cause of TBI in elderly patients, followed by motor vehicle accidents (MVA) [5, 19, 25–27]. It may be postulated that the increase in elderly TBI can be explained by an increase in life expectancy and advances in health care; while physiologically, elderly people are more susceptible to fall.

Fall is a major disease burden worldwide. In a retrospective cohort in India, 40 % of the elderly patients who fell injured their heads, where half of them presented with severe head injury (Glasgow Coma Scale [GCS] < 9) [28]. In the United States (Oklahoma), the rate of elderly fall causing TBI showed 120 % increase over the last decade [18].

Hong Kong, one of the busiest cities in Asia, has a low MVA-related death rate (2.4 per 100,000), which is 25 % of Australian and 15 % of U.S. rates. In Hong Kong, 37 % of MVA-related death was among elderly people over 60 years old. Over 80 % of the deceased across all age groups sustained head injury [29].

## Pathology

Acute subdural hematoma was the commonest pathology for elderly TBI [30], which corresponds with a coroner’s report that subdural hematoma was the commonest lesion of the deceased elderly persons with head injuries [12].

## Comorbidities

Medical comorbidities are found in 40 % of the elderly population [31]. They are more likely to suffer from cardiovascular diseases, and may lead to the overwhelming prescription of anticoagulation and antiplatelet agents, which are discussed in details below.

### Anticoagulants

Almost one fifth of elderly patients older than 65 years old admitted to an American level 1 trauma center with head injury were on warfarin, reflecting the importance of impact of warfarin on management and outcome of head injuries [14].

Warfarin consumption is independently associated with higher mortality among a cohort of 384 TBI patients over 55 years old [32]. Those on warfarin with minor head injuries (GCS ≥ 13) are 2.7 times more likely to suffer from intracranial hemorrhage (ICH), and traumatic ICH was associated with supratherapeutic international normalized ratio (INR) [33].

In a retrospective analysis of 1493 patients with head injury, INR over 4.0 and age over 70 years were associated with higher mortality. Warfarin carries a sixfold increase in TBI mortality [34]. Pieracci et al. [14] compared warfarin users with mean INR 3.3 versus those with suboptimal level, with the former group having a lower GCS score on presentation and higher mortality after head injury. Karni et al. [35] reported similarly that the 30-day mortality was 50 % in warfarinized elderly patients with mean INR 3.0, compared to the mortality of 20 % in nonwarfarinized counterparts. With a mean initial INR of 4.0, warfarin can be even more lethal in elderly TBI, with over 80 % mortality in those with initial minor head injury [36].

Delayed ICH with oral anticoagulant and head injury also were reported among elderly patients [37]. However, routine follow scan in negative first computer tomography (CT) scan of brain in warfarinized patients has a very low yield of 1 % delayed ICH after head injury [22, 38].

New direct coagulation factor inhibitors, such as dabigatran, have suggested superior stroke and systemic embolism prevention with less drug–drug interactions. However, there is still no specific reversal agent and routine laboratory essay to measure anticoagulation level in patients with traumatic ICH [39].

### Antiplatelets

Pre-injury antiplatelet agents were associated with three times higher mortality among patients over 50 years old, where it was due to a functional rather than quantitative deficiency of platelets [26].

Intracranial bleeding progressed in 10 % of elderly TBI patients on clopidogrel. They were three times more likely to be discharged to long-term inpatient facilities and 14 times higher mortality rate as compared with those not on antiplatelets [40].

However, platelet transfusion was shown not beneficial in terms of mortality among patients older than 50 years old with pre-injury antiplatelets who suffered from TBI with ICH [41]. Routine platelet transfusion may even be harmful. Washington et al. [42••] showed that in patients who were on antiplatelets and sustained minor head injury, where the first CT scan showed intracranial hematoma that did not require immediate operative treatment, platelet transfusion had a higher rate of medical complications, including death due to cardiac causes. There was no difference in terms of neurological decline between the transfused and nontransfused group. Fortuna et al. [43] similarly reported higher mortality for patients with pre-injury antiplatelets who received platelet transfusion therapy compared with those who were not transfused. Utilization of commercially available aspirin response test could assess the effect of existing aspirin therapy in TBI patients, thus possibly avoiding unnecessary futile platelet transfusion [44].

### β-Blockers

The relationship with use of β-blockers and outcome in TBI patients is complex. On one hand, some retrospective data suggest that β-blocker users have a lower mortality in elderly patients suffering from severe blunt head injury, hypothesizing that β-blockade may result in attenuation of intracerebral post-traumatic catecholamine-induced vasospasm, decreasing potential for local ischemia [45]. However, in those with severe systemic injuries, β-blockers may mask the systemic response to trauma, leading to higher mortality [21].

### Statins

Preinjury statin use in elderly TBI patients is associated with reduced risk of death and greater likelihood of achieving a good recovery at 12 months postinjury, possibly related to anti-inflammatory and immunomodulatory effects of statins [23].

## Functional Outcome

The overall mortality for elderly TBI patients older than 65 years old was 14 %–55 % [11••, 17–19, 24, 25, 46], and was affected mainly by GCS on admission, followed by age.

GCS on admission, described by Jennett et al. in 1976 [47], had long been known to predict outcome. In a large multicenter epidemiologic study of TBI in China, minor head injury patients (GCS 13–15) had over 90 % survival, where less than 20 % achieved good outcome in severe head injury patients (GCS 3–8) [46]. Several studies also showed severe head injury among elderly led to high mortality, ranging from 68 %–92.5 %, and less than one fifth achieved good outcome at 6 months (Table 1).

**Table 1:** Outcome of elderly traumatic brain injury with severe head injury (GCS 3–8 on admission)

Study	Year	Period of data collection	Age, *y*	Patients, *n*	Mortality, *%*	Good outcome at 6 months*, *%*
Kilaru et al. [48]	1996	1990–1995	65+	40	68	7.5
Hukkloven et al. [49]	2003	1991–1995	65+	101	72	15
Ushewokunze et al. [30]	2004	1990–2000	70+	71	80	1
Gan et al. [19]	2004	1999–2001	64+	36	72.2	16.7
Patel et al. [8••]	2010	1996–2004	65+	669	80.7	
			65–70	137	71.5	
			70–75	147	74.8	
			75–80	160	85	
			80+	225	87	
Bouras et al. [16]	2007	1998–2005	65–74	48	79.2	
			75+	67	92.5	

*Good outcome as defined by Glasgow Outcome Score 4–5

*GCS* Glasgow Coma Scale

Despite the fact that older patients tend to suffer from less severe injuries, elderly age is associated with higher mortality and worse functional status at discharge [3, 15••, 24, 25, 50]. Elderly patients over 70 years old sustaining severe head injury had 80 % mortality and no one made good recovery at 6 months. The outcome was similar in the same center over 10 year’s time. However, the sample size was small [30].

Another multicenter study in Japan reported that good recovery was only achieved in 10 % in those victims over 50 years old, with over 60 % mortality, and 10 % remained in vegetative state. The factor of neurosurgical intervention was not included [51].

Recent large-scale studies suggested that the outcome of elderly TBI is encouraging. Elderly patients over 65 years old had a survival rate of over 80 % at a retrospective cohort data at a level II trauma center in Florida from 2005 to 2008 [11••]. In a large-scale multicenter epidemiologic study of TBI in Eastern China conducted in 2004, around 60 % of TBI patients over 65 years old enjoyed good recovery [46].

Increasing number of elderly individuals are surviving moderate to severe TBI over the past two decades as reported by Ramanathan et al. [15], similar to findings using statewide data collected in Oklahoma [18]. When adjusted for sex, year of TBI, and TBI severity, elderly patients showed similar risk of death following head injury as younger patients [17]. In a 10-year Singapore review, though significantly less than the younger victims, 36 % of elderly with initial GCS over 8 managed to attain good outcome [19].

Inpatient rehabilitation was shown to improve outcome at 6 months even in certain deeply comatose elderly head injury patients, as measured by significant gains on the modified Barthel Index after rehabilitation [52].

### “Younger” Elderly

Neurosurgical unit care, especially in intensive care unit, significantly reduced mortality in those with severe head injury between 65 and 70 years old. Mortality in severe TBI elderly with GCS 6–8 was 47 % and 56 % in patients of 65–70 years old and 70–75 years old respectively, which is much better than the overall mortality of 78.5 % in the overall elderly group [8••].

This is similar with the data from the Greek group [16], that there is a trend of improved survival of the “younger elderly” (65–75 years old) with ICU treatment. Furthermore, the “younger elderly” had a comparable survival with the younger counterparts who are less than 65 years old after surgical intervention in patients with GCS of 8 or higher.

### Surgical Intervention

Overall survival and good recovery following craniotomy in elderly head injury was 30 %–77 %, with GCS over 8 upon admission showing better outcome [7, 20, 31, 53••, 54]. Volume of traumatic ICH greater than 50 ml was associated with poor 1-year outcome [25]. A cohort showed that, with appropriate postoperative medical attention, selected patients older than 80 years old with single traumatic hematoma undergoing craniotomy can similarly return to preinjury functional conditions as their younger counterparts [55•].

On the other hand, decompressive craniectomy does not show significant benefit in older patients after failing maximal medical treatment [10]. At 1 year after decompressive craniectomy, 80 % of elderly patients with severe TBI had poor outcome. Most patients who died had a controlled intracranial pressure (ICP) after surgery and no surgery-related complications. The brain’s greater exposure to minor repetitive insults as age increases, together with the presence of systemic comorbidities such as vasculopathies and neuropathies, likely contributes to the brain’s reduced capacity for recovery and may therefore have an inferior impact on prognosis [9].

## Cognitive Outcome

There is some evidence to suggest that moderate to severe head injury can predispose to development of cognitive decline and even Alzheimer’s disease decades afterwards [56–58]. A 9-year population-based study of elderly patients also showed fall-related TBI predicts earlier onset of dementia and the effect is especially high amongst patients who carry the apolipoprotein E ε4 allele [59]. However, it is not reproducible in other studies, and failed to find an association between history of TBI and dementia [60]. Elderly patients are also more prone to cognitive dysfunction after TBI [61].

Repetitive minor blunt head injuries can cause chronic traumatic encephalopathy at a later stage of life, characterized with memory disturbances, behavioral and personality changes, Parkinsonism, and speech and gait abnormalities. Pathologically, there are extensive tau-immunoreactive neurofibrillary tangles throughout the brain in relative absence of β-amyloid deposits. There are reports that repetitive head trauma also can be associated with development of motor neuron disease [62, 63].

According to a systemic review, prevalence of depression in community-residing elderly adults ranges from 1.8 %–8.9 % in the community and 10 %–25 % in medical and long-term care settings. In the general population with TBI, prevalence rates of depression range from 10 %–42 %, with older age being a significant factor [13, 64]. Imaging study based on magnetic resonance imaging (MRI) suggested the possible role of frontotemporal lobe and basal ganglia pathology in depression after TBI [65]. TBI may increase the risk of disinhibition in elderly patients with dementia [66].

## Animal and Experimental Studies

Researchers have been trying to determine why and how age plays a role in outcome of TBI. Onyszchuk and colleagues [67, 68] studied the effect of trauma to the brain among mice. Aged mice performed worse in general compared with the adult group. The aged group showed slower resolution in brain edema in MRI. Immunostaining showed greater degree in opening in blood–brain barriers and higher degree in neurodegeneration in aged mice. The effects not only affected the acute phase, but also were present 2 months after injury. A similar study comparing both the aged and younger mice was published by Sandhir et al. [69], who focused on the age-dependent response of CCAAT/enhancer binding proteins (CEBP). The aged brain showed a significant increase in both the CEBP β- and δ- protein, which are responsible for glia activation in neurotoxic events and development of Alzheimer's disease.

There have been recent advances in genetic studies related to TBI. Colak et al. [70] identified that several genes were upregulated in injured brains (eg, C1ql2, Cbnl, 5 dc1 Bdnf, MMP9, and Cd47), which are responsible for neuroprotection and nerve repair. Mehan et al. [71] showed that a series of protein isoforms was induced only in juvenile brains but not in the elder brains among mice.

Targeted therapies toward these particular genes have been studied, and age also serves as an important factor in the response of the brain toward the treatment modalities. Nicotinamide was found to be only effective in younger mice to improve functional recovery in TBI [72]. Nogo-A/B, myelin-derived inhibitors to axonal outgrowth, were genetically modified to be negative in experimental mice. The younger mice expectedly had a better outcome, yet showed contrary results in the aged group [73].

**Fig. 1:**
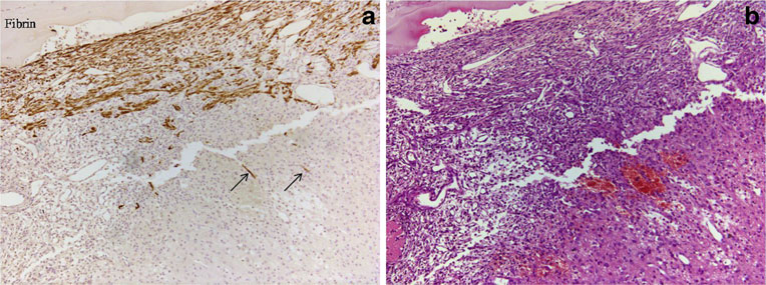
Topical application of mesenchymal stem cells (MSCs) as potential treatment following severe traumatic brain injury (TBI) in rats. MSCs derived from transgenic Sprague-Dawley (SD) rat with green fluorescent protein (GFP) and a thin layer of fibrin were topically applied on the surface of brain with TBI. **a** Immunochemistry staining using anti-GFP (IHC x 100; brown) showed the migration of GFP-MSCs (*arrows*) into parenchymal brain tissue 3 days after topical MSC application. **b** H & E counter-staining (x 100). *IHC* immunohistochemical staining; *H & E* hematoxylin and eosin

Preclinical trials focused on stem cell therapy to traumatic nervous tissue injury are promising, as summarized by Harting and colleagues [74]. Embryonic stem cells (ESCs) derived from the inner mass of blastocyst are capable of producing all tissues of the body [75]. Neural stem cells (NSCs) isolated from subventricular zone and dentate gyrus of hippocampus are multipotent cells with the potential to differentiate into neurons, oligodendrocytes, and astrocytes [76]. Clinical applications of ESCs and NSCs are hindered by ethical controversies, concern of tumor formation, and limited availability respectively. Mesenchymal stem cells (MSCs) can be isolated from bone marrow and adipose tissue, among other sources, and rapidly expanded in vitro without losing their general multipotentiality [77]. MSCs also can differentiate into specific cell lineages under appropriate microenvironment. The brain itself has a limited capacity for self-repair. Preclinical studies have demonstrated that MSCs can facilitate the replacement of injured or nonfunctioning cells and improve the functional recovery after TBI [78–80], although the underlying mechanism remains to be defined. Apart from engraftment and transdifferentiation into neurons and astrocytes or fusion with the host cells, MSCs may stimulate neurogenesis, gliagenesis, and synaptogensis via secretion of neuroprotective/trophic factors as well as modulation of microenvironment inflammatory and systemic immunologic responses [81, 82]. TBI elicits a cascade of pathophysiologic processes leading to neuronal damage. Conventional pharmacologic agents usually target only a single pathway, whereas stem cell–based therapies can execute multiple mechanisms and thus give rise to the hope that MSCs may be able to reverse cellular/subcellular cascade underlying brain damage following TBI. Intravenous and intra-arterial injections are most commonly routes for the administration of MSCs in clinical trials. To date, no adverse clinical complication caused by infusion of expanded autologous MSCs for neurological diseases has been reported [83, 84]. However, few infused MSCs reach the injured lesion of the brain because of the pulmonary first-pass effect [85]. The average diameter of MSCs is smaller than that of pulmonary artery. A recent study demonstrated that topically applied MSCs can migrate to the injured parenchymal tissue in the models of TBI (Fig. 1) [86], and ischemia-reperfusion injury in other somatic organs [87•]. Topical application can offer a direct delivery of millions of MSCs to the brain. Clinical efficacy and feasibility of cell-based therapies for TBI require further investigation in regard to cell types, cell dosage, routes of administration, and transplantation window.

## Conclusions

Elderly TBI has been and will be an important burden to the society with longer life expectancy and an aging population, with fall being the commonest cause. Increasing use of antiplatelet and anticoagulation medication is going to further complicate the condition. Outcome following TBI in “younger elderly” (ie, those less than 75 years old) can be comparable with younger adults with acceptable outcome. With good case selection of minor to moderate head injury in those relatively younger elderly victims, surgical treatment should not be withheld, and traumatic hematoma evacuation may be beneficial among elderly as opposed to traditional thoughts. Current literature lack dedicated studies to this particular group of patients with less severe head injuries. Animal and laboratory studies showed promising explanation of underlying mechanisms of elderly TBI and development of novel treatment.
